# Prognostic Value of the Area of Lung Involved in Severe and Non-Severe Bronchiolitis: An Observational, Ultrasound-Based Study

**DOI:** 10.3390/jcm13010084

**Published:** 2023-12-23

**Authors:** Anna Camporesi, Luigi Vetrugno, Rosa Morello, Cristina De Rose, Stefania Ferrario, Danilo Buonsenso

**Affiliations:** 1 Pediatric Anesthesia and Intensive Care, Buzzi Children’s Hospital, 20154 Milano, Italy; stefania.ferrrario@asst-fbf-sacco.it; 2 Department of Medical, Oral and Biotechnological Sciences, University of Chieti-Pescara, 66100 Chieti, Italy; luigi.vetrugno@unich.it; 3 Department of Woman and Child Health and Public Health, Fondazione Policlinico Universitario “A. Gemelli”, 00168 Roma, Italy; rosa.morello91@gmail.com (R.M.); cristyderose@gmail.com (C.D.R.); danilobuonsenso@gmail.com (D.B.); 4 Centro di Salute Globale, Università Cattolica del Sacro Cuore, 00168 Roma, Italy

**Keywords:** bronchiolitis, Pediatric Intensive Care Unit (PICU), Continuous Positive Airway Pressure (CPAP), superior lung lobes, lung regional differences, bronchial geometry

## Abstract

Background: Point of care lung ultrasound (LUS) has a definite role in viral bronchiolitis when combined with clinical data. Previous data showed a bigger involvement of the superior lung zones in more severe cases. The aim of the present study is to describe whether different lung areas are implicated to different degrees in patients admitted to a Pediatric Intensive Care Unit (PICU) and needing ventilation compared to those with less severe forms. Methods: observational, prospective study. LUS scores of single lung areas and clinical data were collected for all children aged 0–12 months presenting with bronchiolitis to the participating centers and used as covariates for logistic regression having “PICU admission” as outcome. A subsequent analysis was carried out to investigate factors concurring with different lung zones’ involvement. Results: 173 patients were enrolled. Difficulty in feeding, presence of wheezing, SpO_2_ were all risk factors for PICU admission. Superior lung areas’ LUS scores presented higher Odds Ratios for PICU admission and need for ventilation than inferior ones. Age and prematurity concurred in determining their higher LUS scores. Conclusions: Superior lobes’ greater involvement could be favored by the geometrical distribution of relative bronchi, exiting with an acute angle from mainstem bronchi in small children where airway caliber is small and only small volumes of secretions can be occlusive.

## 1. Introduction

Bronchiolitis is a leading cause of morbidity and mortality in children worldwide [1]. Despite being one of the commonest pediatric diseases, uncertainties remain about best diagnostics, treatment strategies and tools to recognize early risk of deterioration. In fact, one of the main challenges that pediatricians face is how to manage a child with a full-blown clinical picture of bronchiolitis but with vital parameters within normal values or borderline, since children with bronchiolitis can deteriorate within hours after initial observation [2]. While observing these children to monitor progression may be the ideal strategy, this is usually not feasible nor sustainable due to a lack of beds in the observation units and not enough healthcare resources. Therefore, prognostic biomarkers are highly needed.

In this context, point-of-care Lung Ultrasound (LUS) has been widely investigated and several papers in different countries have demonstrated that LUS is able to detect low respiratory tract involvement during bronchiolitis, but also that higher LUS scores are associated with more severe clinical presentations [3,4,5,6,7,8,9,10,11]. However, these studies have usually included a small number of patients needing Pediatric Intensive Care Unit (PICU) admission or different respiratory supports. More importantly, studies that combine clinical and LUS data are still lacking. This is an important gap in the field of LUS since it is well established that LUS is a clinical tool and should always be interpreted in combination with clinical findings and clinical suspicion [12,13]. In a previous preliminary report, we tested in a smaller cohort a clinical–ultrasound combined score based on a minimum number of clinical and ultrasound finding, showing that wheezing, difficulty in feeding and superior lobe atelectasis were associated with higher risk of PICU admission and need for ventilation [14]. This greater involvement of upper lung areas in severe cases prompted us to investigate in deeper detail the regional variations between lung areas during severe and non-severe bronchiolitis. The aim of the present study is therefore to describe if different lung areas are implicated to different degrees in patients admitted to PICU and/or needing ventilation compared to those with less severe forms of bronchiolitis.

## 2. Methods

### 2.1. Population

This is an observational, prospective study, conducted between November 2021 and April 2023 in the Pediatric Emergency Departments of the “Gemelli University Hospital” in Rome and of the “Vittore Buzzi Children’s Hospital” in Milano (Italy). All consecutive children aged 0–12 months with a clinical diagnosis of bronchiolitis and admitted to the two centers were enrolled; for each child, demographic and clinical information were collected and saved on a standardized form before the execution of the ultrasound exam.

Patients were excluded from the study if they presented immunodepression, heart diseases, neuromuscular diseases, cystic fibrosis, bronchopulmonary dysplasia (defined according to the perinatal history, dependence on oxygen), positive history of foreign body inhalation, unstable critical conditions that required immediate life-saving procedures and lack of parental consent.

PICU admission was deemed necessary if the patient presented with high supplemental oxygen requirement (FiO_2_ (Inspiratory Fraction of Oxygen) ≥0.5 to maintain SpO_2_ (Peripheral Saturation of Oxygen) ≥92%), need for non-invasive or invasive ventilation, rapidly progressive upper or lower airway disease, apnea observed by a physician or nurse or described as cyanosis and/or loss of consciousness and/or decreased muscle tone [15].

An individual data sheet for the collection of demographic, medical and clinical data, according to the clinical classification of bronchiolitis, was used.

The study was approved by the Ethics Committees of each participating center.

The primary aim of the present study is to describe and compare clinical and LUS characteristics of infants with bronchiolitis needing admission to PICU to those who do not. The secondary aim of the study is to compare regional differences in LUS scores of different lung areas in patients admitted to PICU and children who did not need it, and to describe factors that could be contributing to the risk of a worse LUS score as a total and in different areas.

### 2.2. Lung Ultrasound

Lung ultrasound examinations were performed by in the Emergency Department of both centers, with a high frequency (3–12 MHz) linear probe (Affiniti 70, Philips, Amsterdam, The Netherlands), at the patient’s bedside, using a modified three-zone per hemithorax Bedside Lung Ultrasound in Emergency (BLUE) protocol (described by Lichtenstein [16]). In Milano, the ultrasound examinations were performed or reviewed by A.C. and in Rome they were performed or reviewed by D.B. Both A.C. and D.B. are pediatric emergency physicians, fully trained in lung ultrasound examinations, with extensive experience in the field.

**Figure 1:**
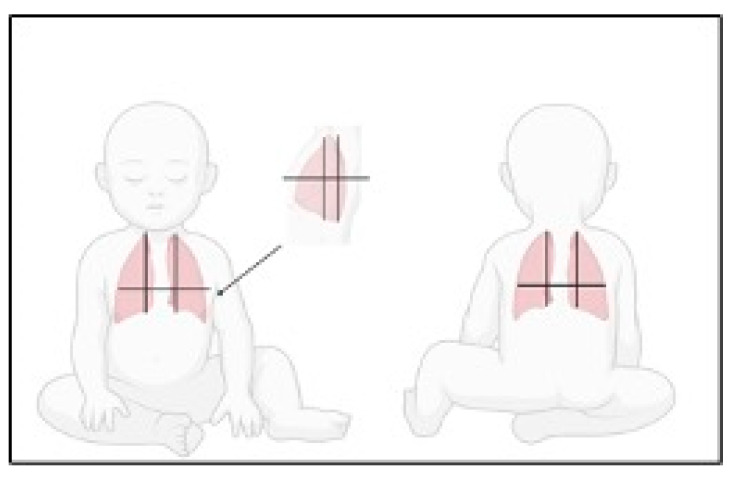
Schematic representation of the lung areas investigated by lung ultrasound (LUS). Chest areas were identified by parasternal, anterior axillary, internipple lines and spine.

For the lung ultrasound scan, the same protocol is followed in both centers, which collaborate habitually in pediatric lung ultrasound research. Each hemithorax is divided into anterior, lateral and posterior zones, and upper and lower zones (divided by the internipple line); the anterior aspect of the chest is identified by the anterior axillary line, the lateral aspect of the chest by the anterior and posterior axillary lines and the posterior aspect of the chest by the posterior axillary line and the spine, not including the scapular area. Children are scanned in a recumbent or semirecumbent position and rolled onto their side to optimize posterior scanning (Figure 1).

The lung areas are therefore classified as follows: Right Anterior Superior (RAS); Right Anterior Inferior (RAI); Left Anterior Superior (LAS) and Left Anterior Inferior (LAI); Right Lateral Superior (RLS) and Right Lateral Inferior (RLI); Left Lateral Superior (LLS) and Left Lateral Inferior (LLI); Right Posterior Superior (RPS) and Right Posterior Inferior (RPI); Left Posterior Superior (LPS) and Left Posterior Inferior (LPI).

**Anterior zones** are grouped and defined as the sum of the Right Anterior Superior, Right Anterior Inferior, Left Anterior Superior and Left Anterior Inferior zones.

**Lateral zones** are defined as the sum of the Right Lateral Superior, Right Lateral Inferior, Left Lateral Superior and Left Lateral Inferior zones.

**Posterior Zones** are defined as the sum of the Right Posterior Superior, Right Posterior Inferior, Left Posterior Superior and Left Posterior Inferior zones.

**Superior Zones** are defined as the sum of the Right and Left Anterior Superior, Lateral Superior and Posterior Superior zones.

**Inferior Zones** are defined as the sum of the Right and Left Anterior Inferior, Lateral Inferior and Posterior Inferior zones.

In order to characterize the LUS patterns, the recently published definitions of the Italian Academy of Thoracic Ultrasound (Accademia di Ecografia Toracica, ADET) [17] were employed:

0: normal A lines;

1: short vertical artifacts and isolated B lines;

2: multiple B lines (B lines with less than half a centimeter to the confluence, remaining identifiable from each other);

3: white lung (subpleural field with various shades of gray/white without distinguishing B lines) and subpleural consolidations smaller than 1 cm;

4: subpleural consolidations bigger than 1 cm.

### 2.3. Statistical Analysis

For the outcome “PICU admission”, a multivariable logistic regression was conducted with the following potential candidate predictors: age, sex, ex-premature status (defined as birth before 36 + 6 weeks of gestational age); under a clinical aspect: symptom duration before medical consult expressed in hours, presence of rhinorrhea, fever, retractions, crackles, wheezing, reduced food intake, SpO_2_; under a microbiological aspect, detection of RSV (Respiratory Syncytial Virus) or multiple viruses; under the ultrasonographic aspect, the single zones’ score, the macro-areas’ score and the total LUS Score according to the previous definition. The goodness-of-fit of the model was tested for every analysis with a Hosmer–Lemeshow test. Possible final models were evaluated comparing their respective Akaike Information Criterion (AIC) and Bayesian Information Criterion (BIC).

Total LUS score as well as macro-areas’ LUS scores were used as dependent variables in multivariable linear regression models using all variables that could, from a theoretical point of view, concur with them as covariates: age (expressed in months), sex, premature status, symptom duration (hours) fever, detection of RSV or multiple viruses in nasal aspirate. Multicollinearity was checked with a Variance Inflation Factor (VIF) test.

Quantitative variables were described by the mean and standard deviation (SD) or median and Interquartile Range (IQR), depending on the variable distribution. Frequencies and percentages were used for categorical variables.

We used a Chi- square test to analyze categorical variables and a Wilcoxon rank-sum test for continuous variables. Statistical significance was designated as a *p* value ˂ 0.05 (two sided). Statistical analysis was performed with STATA v.18 BE (StataCorp, College Station, TX, USA).

## 3. Results

A total of 173 patients were enrolled in the study: 99 in the present winter season 2022–2023 and 74 in the previous season. A total of 89 patients received High Flow Nasal Canula ventilation (HFNC): 41 of them received it in PICU and 48 in ward. A total of 55 patients were admitted to PICU. A total of 52 were ventilated with Continuous Positive Airway Pressure (CPAP): of these, 51 received CPAP while in PICU. Three patients required Invasive Mechanical Ventilation (IMV) (Table 1).

### 3.1. Lung Ultrasound Scores

Scores of the single lung areas were all significantly different between patients who were then admitted to PICU and those who were not, with the exception of the Right Posterior Inferior lung area. Total LUS score also differed significantly between patients admitted to PICU and those who were not (Table 2). Similar results showed the analysis of US scores for patients ventilated with HFNC (where all lobes’ US scores were significantly different except for the Right Posterior Inferior and Left Lateral Inferior zones) and for those ventilated with CPAP, where all lobes’ US scores differed significantly, with the exception of the Right Posterior Inferior zone again.

### 3.2. Logistic Regression for the Outcome PICU

An analysis was conducted for the outcome “PICU admission”. Multivariable logistic regression conducted on clinical data identified three factors as predictors of PICU admission (Table 3): difficulty in feeding, presence of wheezing, SpO_2_. Single area ultrasound scores were added to the model one at a time; they all concurred in PICU prediction, except for the score of the Right Anterior Inferior and Right Posterior Inferior areas. Total US score also was significant in the multivariable analysis.

### 3.3. Logistic Regression for the Outcome HFNC

Univariate and multivariable analysis showed significance for symptom duration and total LUS score and single areas’ LUS score (with the exception of the Left Anterior Inferior, Left Lateral Inferior, Left Posterior Inferior and Right Posterior Inferior areas).

### 3.4. Logistic Regression for the Outcome CPAP

Univariate analysis selected rhinorrhea, difficulty in feeding and SpO_2_ as factors concurring in the outcome “CPAP” in our cohort. In multivariable analysis, the abovementioned factors were tested together with the ultrasound score of one lung area at a time and the following proved significant: Right Anterior Superior, Left Anterior Superior, Right Lateral Inferior and Superior, Left Lateral Superior, Right Posterior Superior areas.

### 3.5. Linear Regression for the Outcome Total Score

Total ultrasound score was considered as the dependent variable for a linear regression including host factors (namely: age, male sex, prematurity, presence of rhinorrhea, symptom duration, fever, detection of RSV or more than one virus in nasal aspirate). The results of the regression are reported in Table 4.

### 3.6. Superior and Inferior Lung Areas

Since inferior lung areas appeared to be less involved in predicting the need for ventilation or PICU admission, a subsequent analysis was conducted dividing the lung into superior lung areas (namely the sum of RAS, LAS, RLS, LLS, RPS and LPS) and inferior lung areas (RAI, LAI, RLI, LLI, RPI, LPI). These two macro-areas were further investigated in their relationship with PICU, HFNC and CPAP as outcomes and in the factors that could concur with them.

**Figure 2:**
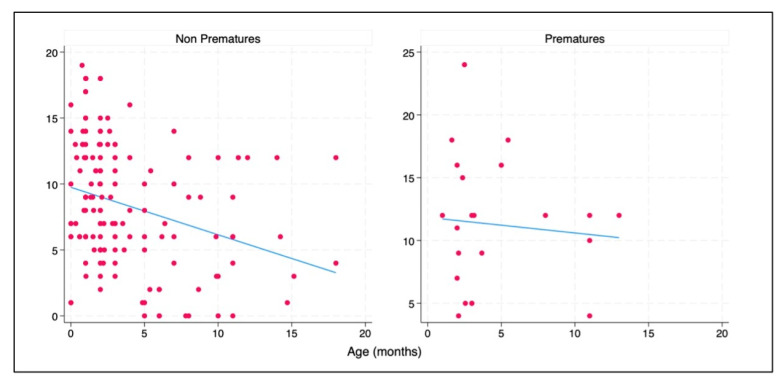
Scatterplot and regression line of effect of age on superior lung areas’ ultrasound score in prematures and non-prematures.

Considering the sum of all superior lobes’ US scores as the dependent variable, a linear regression was conducted, including clinical data as independent variables. Results showed significant correlation with age (negative relationship) and prematurity (positive relationship) in both univariate and multivariable analyses. Given the potential interplay between these two variables, this model was also studied for their interaction, which was not statistically significant. However, it is appreciable how the regression line of the LUS score in superior lung areas is steeper in non-prematures compared to prematures. (Figure 2).

Inferior lung areas’ US scores, on the contrary, did not show similar correlations.

**Figure 3:**
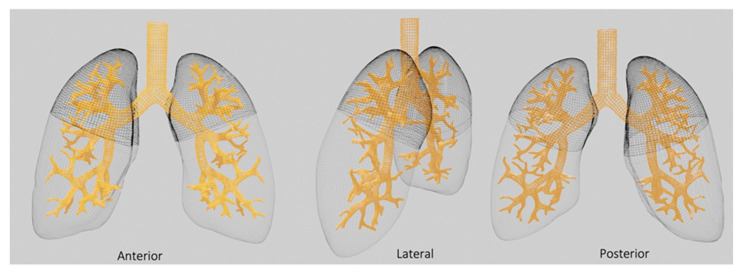
Superior lung areas’ involvement associated with PICU admission, HFNC and CPAP ventilation.

Superior lobes’ scores also concurred more than the inferior ones in predicting risk for PICU admission (OR for superior areas: 1.25, CI: 1.13–1.39 versus OR for inferior areas: 1.15, CI: 1.05–1.26), CPAP (OR superior areas: 1.22, CI: 1.11–1.33, versus OR inferior areas: 1.15, CI: 1.06–1.25) and HFNC ventilation (OR superior areas: 1.16; CI: 1.08–1.25,versus OR for inferior areas: 1.09, CI: 1.02–1.17) (Figure 3).

### 3.7. Posterior, Lateral and Anterior Lung Areas

The sum of the US scores of posterior areas (RPI + RPS + LPI + LPS) was tested with the previous variables and correlated with age (Coeff: −0.24; CI: −0.39–−0.08), symptom duration (Coeff: 0.013; CI: 0.001–0.02) and the presence of rhinorrhea (Coeff: −3.13; CI: −5.5–−0.72).

Anterior areas’ US scores were influenced by prematurity (Coeff: 1.83; CI: 0.28–3.38) and the presence of rhinorrhea (Coeff: −2.23; CI: −4.36–−0.11). Lateral areas’ scores were influenced by prematurity (Coeff: 1.88; CI: 0.33–3.44), age (Coeff: −0.17; CI: −0.30–−0.036) and the presence of rhinorrhea (Coeff: −2.67; CI: −4.79–−0.56).

## 4. Discussion

Our study provides a new insight into the physiopathology of bronchiolitis in the pediatric population, that is, a spotlight on the lung’s regional differences in involvement during the infection and the role of age and co-existing factors such as prematurity. To our knowledge, this is the most comprehensive evaluation of a multi-parameter assessment including clinical, anamnestic and LUS parameters in a cohort of children having different severities of bronchiolitis.

Upper lobes’ greater involvement in more severe cases of bronchiolitis compared to that of the lower lobes is not a new concept. We previously described a population of patients with bronchiolitis studied with lung ultrasound and proposed a combined clinical–ultrasonographic rapid score for detection of severe cases: in this model, the single lung zone most involved in patients then admitted to PICU and requiring ventilation was the Right Posterior Superior Lobe [14]. Our present study reinforces that finding in a bigger sample of patients and extends it to other superior lung areas.

Under physiologic conditions, ventilation has been traditionally postulated to be distributed across the lung on a vertical gradient, where the basal zones are better ventilated than the apical ones, because of regional differences in lung compliance induced by a vertical gradient in lung compression. In this model, the lung is “hanging” at its apex because of a negative pleural pressure and rests on the diaphragm at its base. As the lung is elastic and compressible, alveolar volumes tend to be greatest at the apices and decrease in size down the lung. The smaller alveoli expand more readily because they are on the more compliant portion of their pressure–volume curve, and ventilation is consequently greater at the lung bases [18]. Subsequent studies have then rethought this model, concluding that the distribution of ventilation is determined by a complex interaction of lung and chest wall shapes and by the motion of the lobes relative to each other [19] and that the so-called “vertical gradient” can account only for a part of the differences in ventilation [20,21]. Most physiologists would now agree that the regional distributions of blood flow and ventilation in the lung are determined by both hydrostatic gradients due to gravity and the geometry of the vascular and airway trees. Branching of the airways in humans is irregular [22] and asymmetrical, but in its development, it has probably reached the point where ”the function of the lung can be carried out with minimum entropy production” [23].

These concepts, however, apply to adults and to healthy lungs. The lung of a newborn human at 40 weeks (term) is functional, although it is not simply a smaller version of the adult lung. Also, regional compliance changes with lung injury, and bronchiolitis is such. Some previous papers have described regional variations in ventilation patterns but comparing only ventral to dorsal lung areas, mostly with the aid of complex imaging such as a Computed Tomography (CT) scan [20,21] or Electrical Impedance Tomography (EIT) [24,25]. This technique is innovative, non-invasive and can offer real-time imaging of lung ventilation, but is limited in only being able to offer a partial representation of the lung, such as a “slice” of lung on the coronal plane, without describing regional differences between other lung areas, such as, for example, superior and inferior ones. Lung ultrasound, on the other hand, can offer a point-of-care description of regional ventilation in all planes and zones [26,27] and has a definite role in diagnosing and managing bronchiolitis [4,7,28,29].

Some studies have demonstrated a common involvement of the posterior zones with worse ventilation in many pulmonary pathologies, including bronchiolitis [30]; this is due to the beforementioned gradient in ventilation, which has also the particularity of hitting a population that lives most of the time in a supine position. Anterior areas are better ventilated because of more traction on these areas by intercostal and other contributing muscles.

In small children, the relative and absolute airway diameter is smaller compared to adults, thus, smaller volumes of secretions are needed to occlude them and cause distal dysventilation or atelectasis [29,31]. This potentially applies to all lung areas, but we found a more relevant involvement of superior areas in sicker children. Superior lobes’ greater involvement could be favored by the geometrical distribution of relative bronchi exiting with an acute angle from mainstem bronchi [32,33] that could cause more resistance to airflow, and by the geometrical shape of the thorax in the neonatal age, which is a pyramid with a smaller upper transverse diameter compared to the lower one and starts to reach a “barrel-like” shape, as in adults, after three years of age [34]. In fact, inferior lung areas were found in our study to be less involved in less severe cases, and, in particular, the right posterior inferior one, which also does not receive compression by the heart [31,33]. A better ventilation of the right lung is described in other studies, which used EIT on preterm newborns, both spontaneously breathing and receiving CPAP [24,35].

Among factors influencing regional ultrasound scores, a role was played also by age, symptom duration, rhinorrhea and prematurity, in various combinations. Age showed a negative correlation with the global LUS score; this effect was present in the superior and posterior areas, specifically, but not for the anterior ones. The newborn healthy lung undergoes physiological modifications which are described well with ultrasound [36] and these modifications could concur to our results.

Prematurity was associated with higher scores in the superior lobes, which in turn are associated with more severe disease, as previously discussed. These findings are relatively new and therefore there is not yet a solid existing theory to explain this association between superior lobes’ involvement, severity of bronchiolitis and prematurity. It is possible that a relatively immature lung of a premature child, which may have also been exposed to treatments or procedures such as ventilation that may have had a negative effects on lung parenchyma, may favor the collapse of these zones that may have a more important role in compensating ventilation in young children that are usually in a supine position. Unfortunately, we do not have complete data on degree of prematurity or respiratory support needed at birth and cannot therefore make further speculations on the role of ventilation or gestational age and relative lung immaturity and their potential roles in the patient with bronchiolitis.

Symptom duration had a positive correlation with posterior areas’ US scores but no effect on the other areas, possibly due to a higher probability of further collapse of posterior areas in children experiencing symptoms since longer times, due to the beforementioned characteristics of children’s airways and positioning. Rhinorrhea seems to be a “protective” factor, showing negative correlation with LUS scores, as if patients could present two different phenotypes of the disease or were in two different stages of it: when the upper airways are more involved, then the patient has rhinorrhea, and the lower airways are less involved, as reflected by the lower us score and vice versa.

## 5. Limitations and Strengths

Although our centers have in place the same LUS protocols to be performed in all children with bronchiolitis, the use and indications of HFNC and CPAP could change in each center or even according to the attending physician. However, we believe the strength of our study resides in the relatively high number of patients enrolled and in the reproducibility of the LUS results as only the same two physicians examined all patients.

## 6. Conclusions

In conclusion, we provided insight into regional differences in lung areas’ involvement during bronchiolitis in patients with different severities. The involvement of the superior lung areas had not been studied before and seems to be a predictive factor for more serious diseases. These observations need to be confirmed by further, prospective studies.

## Figures and Tables

**Table 1:** Demographic and clinical data of the population enrolled. HFNC: High Flow Nasal Cannula. CPAP: Continuous Positive Airway Pressure. IMV: Invasive Mechanical Ventilation. CXR: Chest X-ray.

	Total	No PICU	PICU	*p* Value
	N = 173	N = 118	N = 55	
Male sex	90 (52.3%)	61 (52.1%)	29 (52.7%)	0.94
Age (mo)	2.1 (1.0–5.0)	2.2 (1.0–5.0)	2.0 (1.0–6.4)	0.86
Prematurity	23(13.3%)	12(10.2%)	11(20.0%)	0.076
Recent COVID	2 (2%)	0 (0%)	2 (7%)	0.041
Symptom duration (Hours)	72.3 (49.8)	68.4 (42.6)	80.9 (62.1)	0.13
First episode	152 (87.9%)	109 (92.4%)	43 (78.2%)	0.008
Rhinorrhea	161 (93.1%)	118 (100.0%)	43 (78.2%)	<0.001
Difficulty in feeding	132 (76.3%)	80 (67.8%)	52 (94.5%)	<0.001
Home therapy	36 (23.4%)	29 (24.6%)	7 (19.4%)	0.52
Symptoms and signs				
Crackles	160 (92.5%)	115 (97.5%)	45 (81.8%)	<0.001
Wheeze	40 (23.1%)	15 (12.7%)	25 (45.5%)	<0.001
Retractions	157 (90.8%)	102 (86.4%)	55 (100.0%)	0.004
Fever	65 (37.6%)	44 (37.3%)	21 (38.2%)	0.91
SpO_2_				<0.001
≥96%	48 (27.7%)	41 (34.7%)	7 (12.7%)	
93–95%	39 (22.5%)	30 (25.4%)	9 (16.4%)	
<92%	85 (49.1%)	47 (39.8%)	38 (69.1%)	
Missing	1 (0.6%)	0 (0.0%)	1 (1.8%)	
RSV	125 (82.8%)	82 (84.5%)	43 (79.6%)	0.44
Multiple Viruses	41 (35.0%)	32 (38.6%)	9 (26.5%)	0.21
Treatments				
Oxygen (low flow)	138 (80.2%)	87 (73.7%)	51 (94.4%)	0.002
Antibiotics	54 (31.2%)	22 (18.6%)	32 (58.2%)	<0.001
Hypertonic saline	8 (4.6%)	3 (2.5%)	5 (9.1%)	0.056
Bronchodilators	50 (28.9%)	17 (14.4%)	33 (60.0%)	<0.001
Steroids	52 (30.2%)	16 (13.6%)	36 (66.7%)	<0.001
Epinephrine, nebulized	18 (10.4%)	2 (1.7%)	16 (29.1%)	<0.001
HFNC	89 (51.4%)	48 (40.7%)	41 (74.5%)	<0.001
CPAP	52 (30.1%)	1 (0.8%)	51 (92.7%)	<0.001
IMV	3 (1.7%)	0 (0.0%)	3 (5.5%)	0.010
CXR	66 (39.1%)	19 (16.2%)	47 (90.4%)	<0.001

**Table 2:** Differences in regional and global lung ultrasound scores between patients admitted to PICU and those who were not. Data are expressed as median (IQR).

	Total	No PICU	PICU	*p*-Value
	N = 173	N = 118	N = 55	
Right Anterior Inferior	1.0 (0.0–3.0)	1.0 (0.0–2.0)	1.0 (1.0–3.0)	0.004
Right Anterior Superior	1.0 (0.0–3.0)	1.0 (0.0–2.0)	2.0 (1.0–3.0)	<0.001
Left Anterior Inferior	1.0 (0.0–2.0)	1.0 (0.0–2.0)	2.0 (1.0–3.0)	0.002
Left Anterior Superior	1.0 (0.0–2.0)	0.0 (0.0–1.0)	1.5 (0.0–2.0)	<0.001
Right Lateral Inferior	1.0 (0.0–2.0)	0.0 (0.0–1.0)	1.0 (1.0–2.0)	<0.001
Right Lateral Superior	1.0 (0.0–2.0)	0.0 (0.0–1.0)	2.0 (1.0–2.0)	<0.001
Left Lateral Inferior	1.0 (0.0–3.0)	0.0 (0.0–2.0)	1.5 (1.0–3.0)	<0.001
Left Lateral Superior	1.0 (0.0–2.0)	0.0 (0.0–1.0)	2.0 (1.0–2.5)	<0.001
Right Posterior Inferior	2.0 (1.0–3.0)	2.0 (0.0–3.0)	2.0 (1.0–3.0)	0.17
Right Posterior Superior	3.0 (1.0–3.0)	2.0 (1.0–3.0)	3.0 (2.0–3.0)	0.018
Left Posterior Inferior	2.0 (1.0–3.0)	1.0 (0.0–3.0)	2.5 (1.0–3.0)	0.012
Left Posterior Superior	2.0 (1.0–3.0)	2.0 (0.0–3.0)	2.0 (2.0–3.0)	0.017
Total Score	17.0 (12.0–23.0)	15.0 (9.0–20.0)	22.5 (17.0–28.5)	<0.001

**Table 3:** Results of multivariable analysis for the outcome “PICU admission”. OR: Odds Ratio. CI: Confidence Interval.

PICU	OR	*p* Value	95% CI
Difficulty in feeding	5.49	0.017	1.36–2.21
Wheeze	12.9	0.000	4.34–38.35
SpO_2_	1.94	0.028	1.07–3.52
Total lung ultrasound score	1.13	0.000	1.06–1.19

**Table 4:** Results of multivariable linear regression for the outcome “total ultrasound score”. CI: Confidence Interval.

Total Score	Coefficient	*p* Value	95% CI
Age (months)	−0.51	0.002	−0.83–−0.019
Prematurity	3.89	0.043	0.12–7.66
Rhinorrhea	−7.37	0.005	−12.48–−2.25

## Data Availability

Data can be made available upon reasonable request.
